# Translating clinical findings to the legal norm: the Defendant’s Insanity Assessment Support Scale (DIASS)

**DOI:** 10.1038/s41398-019-0628-x

**Published:** 2019-11-07

**Authors:** Giovanna Parmigiani, Gabriele Mandarelli, Gerben Meynen, Felice Carabellese, Stefano Ferracuti

**Affiliations:** 1 Department of Human Neurosciences, “Sapienza” University of Rome, Rome, Italy; 2 Section of Criminology and Forensic Psychiatry, University of Bari Aldo Moro, Department of Interdisciplinary Medicine, Bari, Italy; 3 Willem Pompe Institute for Criminal Law and Criminology, Utrecht University, Utrecht, The Netherlands; 4 Faculty of Humanities, Vrije Universiteit Amsterdam, Amsterdam, The Netherlands

**Keywords:** Scientific community, Human behaviour

## Abstract

Insanity definition and the threshold for satisfying its legal criteria tend to vary depending on the jurisdictions. Yet, in Western countries, the legal standards for insanity often rely on the presence of cognitive and/or volitional impairment of the defendant at crime time. Despite some efforts having been made to guide and structure criminal responsibility evaluations, a valid instrument that could be useful to guide forensic psychiatrists’ criminal responsibility assessments in different jurisdictions is lacking. This is a gap that needs to be addressed, considering the significant forensic and procedural implications of psychiatric evaluations. In addition, differences in methodology used in insanity assessments may also have consequences for the principle of equal rights for all citizens before the law, which should be guaranteed in the European Union. We developed an instrument, the Defendant’s Insanity Assessment Support Scale (DIASS), which can be useful to support, structure, and guide the insanity assessment across different jurisdictions, in order to improve reliability and consistency of such evaluations.

## Introduction

Insanity evaluations are among the most complex and controversial mental health assessments that psychiatrists and psychologists perform^1,2^. A forensic evaluator is expected to perform a retrospective evaluation of the defendant’s state of mind at the time of crime, to ascertain the presence of a mental disease or defect and to further verify the existence of a possible relationship between that state of mind and the criminal behavior. In case such a relationship exists, its impact on the defendant’s responsibility is further evaluated, and the conclusions will be used by the judge to assess the defendant’s legal responsibility. Depending on the jurisdiction, the psychiatrist’s task and the threshold for satisfying legal criteria for insanity, as well as the definition of insanity itself, may vary.

In Western countries, the legal standards for insanity often rely on the presence of cognitive and/or volitional impairment of the defendant at crime time^3^. In the Anglo-American systems the most acknowledged standards are the M’Naghten Rule (M’Naghten’s Case, 10 Cl. & Fin. 200, 8 Eng. Rep. 718 (H.L. 1843)), and the The Model Penal Code’s test, also known as the American Law Institute (ALI) standard^4^. The M’Naghten Rule focuses on the cognitive component, and states that a defendant is not found responsible if, due to a mental disorder, he did “*not know the nature and quality of the act he was doing; or if he did know it, that he did not know what he was doing was wrong*”. The Model Penal Code, meanwhile, comprises a cognitive as well as a behavioral component, and states that “*a person is not responsible for criminal conduct if at the time of such conduct as a result of mental disease or defect he lacks substantial capacity either to appreciate the criminality (wrongfulness) of his conduct or to conform his conduct to the requirements of the law”*. In those cases where insanity is ascertained, the defendant would be adjudicated either not guilty by reason of insanity (NGRI or NGI) or guilty but not criminally responsible, depending on the legal system^5^.

A negative attitude toward the insanity defense has been reported, and it has been found to be associated with juror judgments^6,7^. A common perception by the lay public is that the insanity defense is overused and might allow criminals to avoid punishment, a belief that appears to entail an implicit distrust regarding the underlying forensic mental health evaluations^6–10^. Such a perception, however, might be inaccurate in view of the existing data. For instance, in a dated pioneering study, it was found that insanity pleas are raised in about 1% of felony cases and proving successful only in about 28% of those cases^11^. Nevertheless, a more recent study from our group showed 42% of insanity judgments among evaluated defendants^12^. These differences are mainly due to different legal thresholds for admitting psychiatric evidence in criminal cases that vary broadly.

A possible source of distrust lies however in the frequent disagreement among experts, with a recent meta-analysis showing that forensic evaluators disagreed 25–35% of the time^13^. This may be associated with the intrinsic limits of psychiatric diagnosis, the different and non-standardized evaluation methodologies^2,14^, and with the longitudinal variability of psychiatric symptoms, implying that evaluations carried out at different times can lead to different conclusions on the same case. As a consequence, expert evaluation has often been considered a “battle of the experts” rather than accurate, objective, and reliable testimony on the defendant’s criminal responsibility—in particular in adversarial legal systems^15^. The absence of biological markers available to guide forensic psychiatric evaluations, and the relative scarcity of reliable and diagnostic tools to guide such assessments, might also account for discrepancies between expert testimonies. In addition, the paucity of research on insanity evaluations implies poor empirical support underlying such assessment^2^. Finally, the dialectic of the criminal trial must be acknowledged, where different parties plead their case, which entails the possibility of different opinions, which the court or jury weighs. At the same time, we should be cautious in interpreting this finding^13^, since this analysis concerns those cases that went to trial. In some jurisdictions, when the experts agree, the cases may not go to trial.

Efforts have been made to guide and structure criminal responsibility evaluations, for example the American Academy of Psychiatry and Law published practice guidelines for insanity defense evaluations^5^, which mainly deal with the steps and information needed to perform them. Among the forensic assessment instruments to assist the criminal responsibility evaluation, are the “Mental State at the Time of the Offense Screening Evaluation” (MSE)^16^ and the “Rogers Criminal Responsibility Assessment Scales” (R-CRAS)^17,18^. The MSE is a semi-structured interview to screen out defendants whose criminal conduct clearly was not caused by significant mental abnormality^19^. The R-CRAS was developed from the American Law Institute’s criteria for the insanity defense, and is composed of 25 items organized into 5 scales assessing: reliability (including malingering), organic factors, psychopathology, cognitive control, and behavioral control^18^. In addition, some theoretical models have been proposed to guide the insanity evaluation^20–24^. However, to the best of our knowledge, a valid instrument that could be useful to guide mental health experts in criminal responsibility assessments in different jurisdictions, is lacking. This is a lacuna that deserves to be addressed, considering the significant forensic and procedural implications of psychiatric evaluations. Basically, two types of errors may occur:

1. An (insane) defendant who is mistakenly considered to be accountable for a crime, despite the presence and influence of a significant psychiatric disorder on his criminal behavior, will find himself/herself punished for a crime for which he should not be held responsible. No justice is being done. Moreover, he could enter the penitentiary system with fewer possibilities to be treated for his disease.
2. A (sane) defendant who is erroneously acquitted because considered insane at the moment of the crime will not be punished for a crime he/she should have been held responsible for. No justice is being done. In addition, he/she will enter a forensic psychiatric system, by using treatment resources that are usually limited.

Differences in methodology used in insanity assessments may also have consequences for the principle of equal rights for all citizens before the law, which should be guaranteed in the European Union.

Furthermore, the availability of a tool that can be used in forensic psychiatric practice could facilitate the exchange of empirical data in research across different jurisdictions and disciplines, thus implementing the evidence that could be of empirical support. Some efforts to shed light on the processes underlying forensic evaluators’ decision-making during the insanity assessment have already been made^12,25,26^.

The aim of this study was to develop the Defendant’s Insanity Assessment Support Scale (DIASS), an instrument, which can be useful to support, structure, and guide the insanity assessment across different jurisdictions, in order to improve reliability and consistency of such evaluations.

## Methods

### The development of the Defendant’s Insanity Assessment Support Scale (DIASS)

The authors initially reviewed insanity criteria applied in different countries^3,5,27–29^. In the second phase, the authors used the clinical model of competence to consent to treatment, with some adaptations to the legal field, as a conceptual framework to guide the evaluations of insanity^24,28^. The model refers to capacity to consent to treatment as a multidimensional construct that relies on several abilities, i.e., understanding information, evaluating information, rational reasoning, and the capacity to express a clear choice^30^. It was used because it proved to be a theoretical model on decision-making with wide experimental empirical evidence on patients, specifically on patients affected by mental disorders^31–38^ and cognitive dysfunctions^28,39–45^. This decision-making capacity model could straightforwardly be adapted to the forensic evaluation of criminal responsibility regarding the understanding dimension (wrongfulness of the act: legal and moral aspects of the act), the appreciation dimension (the nature of and possible options in the situation; e.g., in terms of threat, danger, and risks), and the reasoning dimension (concerning consequences in terms of pros and cons, etc.). The adaptation of the “expression of a choice” dimension to the legal field, required a deep revision of the original scale’s concept and the introduction of a “behavioral component”.

Despite the fact that not all legal systems consider the behavioral dimension as relevant for assessing criminal responsibility, inhibitory control and executive functioning often play a significant role in the forensic evaluations. This view is also supported by neuroscientific studies showing the presence of cerebral abnormalities in violent offenders, especially prefrontal dysfunctions^46–53^. To what extent these data can be relevant in a forensic psychiatric setting is still a matter of discussion, despite the fact that a neurolaw perspective is receiving growing attention in the last few years^54^.

To integrate both cognitive and control issues, we have structured the Defendant’s Insanity Assessment Support Scale (DIASS), based on two components: the first component concerns epistemic factors examining the defendant’s knowledge/understanding and appreciating at the time of the offense. The second component concerns behavioral control-related factors, referring to the defendant’s reasoning processes and control of voluntary motor activity, at crime time.

The preliminary DIASS was then reviewed by experts in three relevant fields: forensic psychiatry (JH), philosophy (SR), and law (GD). The DIASS is shown in Supplementary Appendix.

### The Defendant’s Insanity Assessment Support Scale (DIASS)

The DIASS has been developed based on a wide view of (competent) decision-making, which reflects core issues relevant to legal insanity in many jurisdictions. The DIASS (see Supplementary Appendix) comprises nine binary items (present/absent) grouped into four dimensions: “Knowledge/understanding of the crime” (3 items), “Appreciating of the crime” (1 item), “Reasoning” (3 items), and “Control of voluntary motor activity” (2 items). The first two dimensions refer to the “Epistemic component”, while the third and fourth dimensions refer to the “Control component”. These dimensions refer to the mental state of the accused at the time of the crime. At the end of the scale, there is a box referring to the final judgments on the Epistemic and Control components, which are scored on a three-point scale (intact, partially compromised, and compromised). After having analyzed each subdimension of the Epistemic component (in those countries based on the M’Naghten rule) or of both the Epistemic and Control components (Model Penal Code), the evaluator can indicate their total or partial score, in order to reach his/her final judgment regarding the defendant’s criminal responsibility. The DIASS is an instrument that should be used only after having examined all the legal and health documentation of the defendant, as well as after having carried out the clinical evaluation. It is basically meant to apply the clinical findings to the relevant law in the jurisdiction where the trial is taking place.

**Fig. 1:**
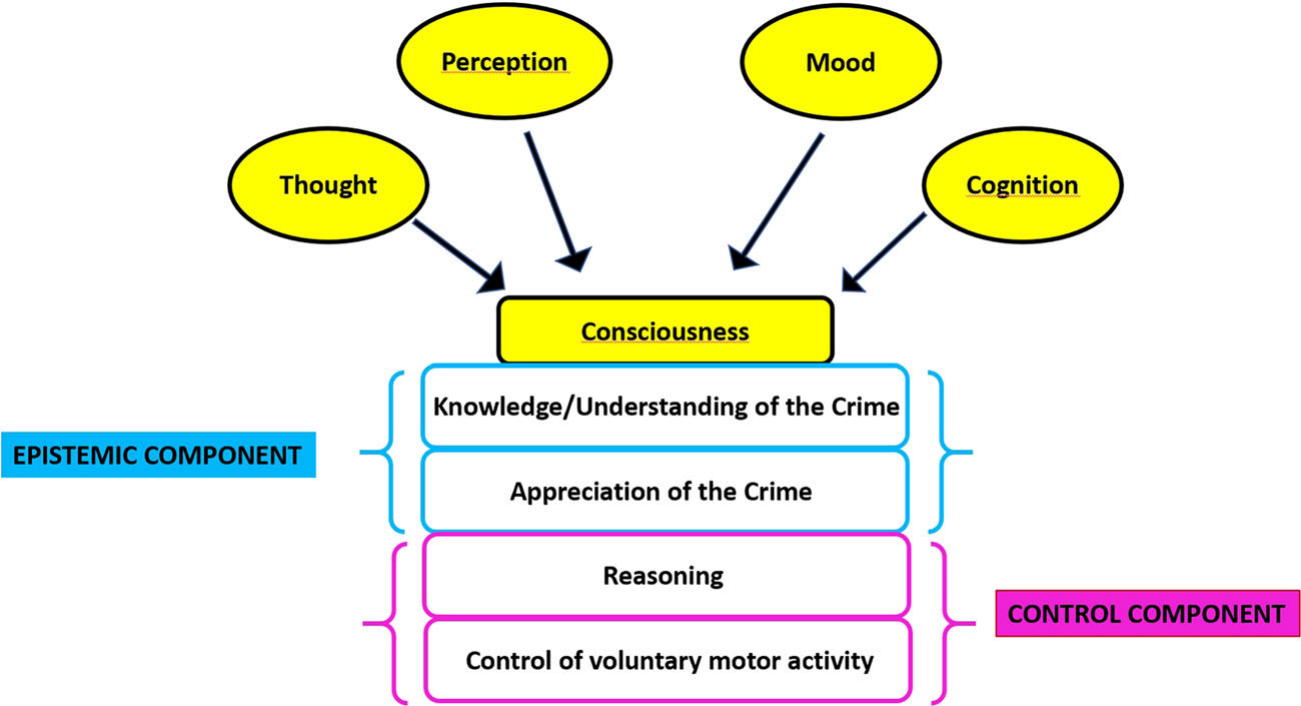
As shown in the figure, psychopathological symptoms, such as thought disorders, perceptual disrurbances, mood alterations, and cognitive dysfunctions can influence all the four dimensions of the DIASS

All the four dimensions of the DIASS can be influenced by psychopathological symptoms, including thought disorders (disorders of the form of thought such as pressure of thought, fight of ideas and logorrhea, circumstantiality, perseveration, thought blocking, and disorders of the content of thought such as delusions), perceptual disturbances (such as illusions and hallucinations), mood alterations (such as depression, excitement, and dysphoria), and cognitive dysfunction (such as attention deficits, memory dysfunctions, impaired reasoning abilities, and executive dysfunctions) (see Fig. 1). For example, a person affected by a paranoid delusion may commit a crime believing that his actions are acts of self-defense, proportionate to the threat (in this case the thought disorder has an impact on the knowledge–understanding of the nature (wrongfulness) of the act). A subject hearing commanding voices from God may believe that his crime is justified, despite it being against the objective moral standard (in this case the perceptual disturbances affect the appreciation of the subjective moral standard). An individual affected by a bipolar disorder in a manic phase may not have the capacity to properly reason about the consequences of his actions in terms of pros and cons (in this case the mood disorder influences the reasoning dimension). Finally, a person affected by a frontotemporal dementia, may have no capacity to inhibit his unlawful action (in this case the cognitive disfunction has an impact on the control of voluntary motor activity). However, among these four groups of psychopathological symptoms, we believe that the cognitive dysfunction deserves a consideration on its own; in those cases in which the subject’s IQ is particularly low or the individual is affected by moderate or severe dementia, it can in fact have an influence on all the four dimensions of the DIASS.

Regarding the applicability of the DIASS in different countries, as legal insanity standards are likely to refer not to all of these components, depending on the specific criteria in a particular jurisdiction, the relevant ones can be selected and evaluated. For example, if the M’Naghten Rule applies, the element of knowledge would be relevant. If the Model Penal Code test is in use, the elements of appreciation (one of the epistemic components) and control (behavioral control) are relevant. The evaluator should use the components of the DIASS that are reflected in the legal criterion relevant to the particular jurisdiction in which he or she evaluates a defendant. For those legal systems where no explicit criteria have been formulated to determine the legally relevant impact of a disorder, such as the Netherlands, we deem that it may be helpful to consider all the components of this tool in order to arrive at an opinion about a defendant’s legal insanity.

The insanity criteria require a mental illness, or a physical disease that has an impact on the defendants’ mental functioning. Different terms have been used in the legal tests: for instance, M’Naghten refers to “disease of the mind”, while the Model Penal Code standard mentions a “mental disease or defect”, the Italian penal code refers to “mental infirmity”. Other standards in different jurisdictions may use alternative languages and terms. The DIASS does not define the criteria for that component of the insanity standard, as it focuses on a functional approach.

Finally, even though the tool is developed to support the expert’s evaluations of legal insanity, the ultimate decision is—depending on the jurisdiction—up to the judge or jury.

## Conclusion

The DIASS has been developed as a guide and a support tool to promote the quality and transparency of insanity evaluation. It is meant to facilitate the application of the clinical findings to the relevant legal context of the jurisdiction where the trial is taking place and it can be used to formulate one’s expert opinion. Depending on the insanity criteria that are relevant in a specific jurisdiction, the evaluator can choose whether to use only one or both the DIASS components. The instrument represents a step toward some standardization that will hopefully promote the exchange of ideas and research findings across jurisdictions and disciplines. This would be a valuable development for an area that is of considerable medical, legal, and societal importance, but that regrettably continues to be understudied.

The use of the DIASS in forensic psychiatric evaluations can be an initial step toward a reduction of the heterogeneity in methodology between countries, which is in line with the principle of equal rights for all citizens before the law, which should be guaranteed in the European Union.

Finally, the proposed instrument is compatible with neuroscience, as the evaluation of the epistemic and behavioral components can be informed by a growing body of neuroscientific data. This will make it possible to perform assessments that are supported by neuroscientific views and findings.

## Supplementary information

Appendix A
