# Supplementary material for: Translating clinical findings to the legal norm: the Defendant’s Insanity Assessment Support Scale (DIASS)
Source: Transl Psychiatry. 2019 Nov 7;9:278. doi: 10.1038/s41398-019-0628-x (PMC6838307; doi:10.1038/s41398-019-0628-x)
Supplement: Supplementary file 1 — Appendix A [file 41398_2019_628_MOESM1_ESM.docm]

**DEFENDANT’S INSANITY ASSESSMENT SUPPORT SCALE (DIASS)**

**Instruction**

The DIASS is a tool developed to support psychiatrists and forensic psychologists regarding the evaluation of a defendant's legal insanity. It consists of two components: an Epistemic component and a Control component. Depending on the legal standard in which it is used, only the epistemic component (e.g., in jurisdictions using the M'Naghten rule) or both epistemic and control components (e.g., in jurisdictions using the Model Penal Code's test) can be used.

The DIASS must be used by the expert only after having analyzed all the relevant legal and health documentation of the accused, as well as after having carried out the clinical evaluation and having verified the presence of a relationship between the alteration and the crime.

The evaluator indicates the presence or absence of the relevant abilities in the scale by referring to the mental state of the accused, reconstructed at the time of the crime.

1. **Epistemic component^[[1]](#footnote-1)^ (Knowledge / Understanding, Appreciating)**

**Knowledge^[[2]](#footnote-2)^ / Understanding^[[3]](#footnote-3)^**

*A1. Crime context.* At the time of crime, did the defendant severely misinterpret the situation (space, time, environment)?

Example 1: a defendant who kills his son because he suffered from a delusional disorder that made him believe that an impostor had taken his place and would have killed him (disorientation with respect to people).

Example 2: a defendant affected by PTSD who attacks a person thinking to be in a complete different/traumatic situation (dissociation of consciousness)

*A2. Nature of the criminal act*. At the time of the crime did the defendant possess the cognitive and perceptive ability to understand the nature and the quality of the criminal act?

Example 1: a defendant with frontotemporal dementia who throws his dog out of the window on the believing it can fly.

Example 2. A defendant affected by schizophrenia who causes the death of her newborn child due to severe malnutrition due to a diet based only on vegetables to purify him

*A3. Anti-juridical value of the crime and moral standard^3^.* At the time of the crime was the defendant capable of understanding that his/her actions were against the law? At the time of the crime was the defendant capable of acknowledge his/her action was morally wrong or in contrast to the moral beliefs and principles generally accepted in his community?

Example 1: a defendant suffering from a psychotic disorder who believes his boss is persecuting him and going to attack him and that he is justified to defend himself against his boss.

Example 2: a defendant affected by erotomanic delusion who started harassing his general practitioner with continuous visits and offensive and sexual phone calls believing that he was courting her

**Appreciating^[[4]](#footnote-4)^**

*B1. Applicability of the subjective moral standard to one's own behaviour*. At the time of the crime was the defendant capable of appreciating his/her action was morally wrong or in contrast to the moral beliefs and principles he/she used to accept and apply?^[[5]](#footnote-5)^

Example 1: a defendant affected by a religious delusion who poisoned all his family believing to having been sent by God to kill the impure. He knows that it is forbidden to poison other people, but he believes to be justified in doing so by God.

Example 2: a defendant affected by schizophrenia killing her child to protect him from demons that will otherwise torment her and the child

1. **Control component**

***Reasoning***

*C1. About the possibility of non-acting or possibility of alternative choice of action.* Was the defendant’s mental state at the time of crime compatible with the perception of possibility of non-acting?

Example 1: a defendant who committed a crime under the effect of auditory hallucinations (commanding voices) that threatened him with eternal suffering.

Example 2: a defendant affected by a major depressive disorder with a delusion of ruin who kills his son who has been diagnosed with an intellectual disability to spare him from a life of suffering and then tries to kill himself

*C2. About consequences (pros and cons)^[[6]](#footnote-6)^.* Was the defendant’s mental state at the time of crime congruent with the possibility to reason about consequences of action in terms of risks and benefits, as well as to weight different options?

Example 1: a defendant who is a man of principles, affected by bipolar disorder who, at the time of crime, was suffering from manic disorder and starts sexually harassing a woman he just met on a bus because 'love is the most important thing on earth'.

Example 2: a defendant affected by bipolar disorder in a manic phase who causes its company to fail with a series of risky investments because he overrides the benefits and underestimates the risks

*C3. Integration of relevant information.* Was the defendant’s mental state at the time of crime congruent with the possibility to decide based on a logical and rational thinking?

Example 1: a defendant affected by schizophrenia with severely disorganized behaviour who, in a public parking, tries to steal a motorcycle under the eyes of all.

Example 2: a person with a (very) low IQ or advanced dementia may have serious problems processing the information.

**Control of voluntary motor activity:**

*D1. Ability to inhibit one’s own behaviour.* At crime time was the defendant capable to inhibit his/her behaviour?

Example 1: a defendant affected by severe intellectual disability who saw a jogger on the street and suddenly stopped and assaulted the woman. After having beaten and raped the woman near a main road with a high probability of being seen, he threw the body off a cliff.

Example 2: a defendant affected by frontotemporal dementia who sexually harasses his domestic worker

*D2. Ability to program, organize, finalize the action.* At the time of the crime was the defendant able to program, organize and finalize the act?

Example 1: a defendant affected by schizophrenia and executive dysfunctions who to extinguish a cigarette sets fire to the ward where he is hospitalized

Example 2: a defendant affected by dementia who takes the highway on the wrong side and makes a car accident

**Epistemic component and Control component final judgements:**

After having analysed each subdimension of the Epistemic component or of both the Epistemic and Control components (depending on the jurisdiction), the evaluator reaches his/her final judgement regarding the defendant’s criminal responsibility.

| **Defendant’s Insanity Assessment Support Scale (DIASS)** | | |
| --- | --- | --- |
| Defendant’s mental state evaluation at crime time | Present | Absent |
| 1. **Epistemic Component** |  |  |
| *Knowledge / Understanding* |  |  |
| A1. Crime context | □ | □ |
| A2. Nature of the criminal act | □ | □ |
| A3. Anti-juridical value of the crime/objective moral standard | □ | □ |
|  |  |  |
| *Appreciation of the criminal behavior* |  |  |
| B1. Subjective moral standard | □ | □ |
|  |  |  |
| 1. **Control component** |  |  |
| *Reasoning* |  |  |
| C1. About possibility of non-acting/alternative choices | □ | □ |
| C2. About consequences (pros and cons) | □ | □ |
| C3. Integration of relevant information | □ | □ |
|  |  |  |
| *Control of voluntary motor activity* |  |  |
| D1. Ability to inhibit one’s own behavior | □ | □ |
| D2. Ability to program, organize, finalize the action | □ | □ |

| 1. **Epistemic component** | 1. **Control component** |
| --- | --- |
| □ Intact  □ Partially compromised  □ Compromised | □ Intact  □ Partially compromised  □ Compromised |

1. The term epistemic refers to knowledge and appreciation, it is derived from philosophy”. (see also Meynen 2016) [↑](#footnote-ref-1)
2. Here we are referring to the defendant’s attitude, his/her ability to know/acknowledge (which for example can be excluded in a serious intellectual disability). We are aware that “*ignorantia legis non excusat*”. [↑](#footnote-ref-2)
3. Understanding represents a deeper form of knowledge as it allows to draw appropriate inferences from answers, while knowledge results from mere acquisition of information (Sinnott-Armstrong and Levy 2011 pag 314) [↑](#footnote-ref-3)
4. Appreciation refers to the individual ability to have a factual and rational understanding of the harmfulness of an act (e.g., a bullet on penetrating the body can cause death) but also implies the involvement of an evaluational component and requires more than merely drawing out abstract inferences. It implies not only cognition and inference but also an emotional component. As Sinnot-Armstrong and Lewy write: “Emotion is necessary for the kind of capacity to appreciate that is required for moral responsibility and therefore should be required for criminal responsibility”; and “On this view, a defendant does not appreciate the wrongfulness of an act if, for example, she does not "internalize the enormity" of the criminal act” (Sinnott-Armstrong and Levy 2011 pag 315) [↑](#footnote-ref-4)
5. Here we are referring to the evaluation of subjective moral standards (for example a defendant believed to be morally justified to do what he did, despite knowing these acts were against the common standard of morality, because of the presence of commanding voices from God) [↑](#footnote-ref-5)
6. Here we refer not only to negative consequences (wrongfulness) but also to benefits (economical, hedonistic, the possibility to avoid responsibilities, etc,) [↑](#footnote-ref-6)
